# Impact of low-temperature, overcast and rainy weather during the reproductive growth stage on lodging resistance of rice

**DOI:** 10.1038/srep46596

**Published:** 2017-04-19

**Authors:** Fei Weng, Wujun Zhang, Xiaoran Wu, Xia Xu, Yanfeng Ding, Ganghua Li, Zhenghui Liu, Shaohua Wang

**Affiliations:** 1 National Engineering and Technology Center for Information Agriculture/Key Laboratory of Crop Physiology and Ecology in Southern China/Jiangsu Collaborative Innovation Center for Modern Crop Production, Nanjing Agricultural University, Nanjing 210095, China; 2 Chongqing Academy of Agricultural Sciences/Chongqing Ratooning Rice Research Center, Chongqing 402160, China

## Abstract

The objectives of this study were to explore the mechanism by which the lodging resistance of the rice population during the late growth period responds to low-temperature, overcast and rainy weather during the reproductive growth stage. Field experiments were conducted using indica rice Yliangyou2 (lodging-resistance variety), IIyou084 (lodging-susceptible variety) and japonica rice Wuyunjing23 (lodging-resistance variety) and W3668 (lodging- susceptible variety) in 2013 (high temperature and strong radiation during the rice reproductive growth stage), 2012 and 2014 (low temperature and weak radiation during rice reproductive growth stage). The results showed that the length of the basal internodes and the height of the gravitational centres were greater in plants grown in 2014. Dry weight of basal culms, culm diameter, lignin content and total content of structural carbohydrates (lignin and cellulose) in basal internodes were reduced in these plants, causing a significant reduction in the bending stress and lodging resistance of the rice stems. Low-temperature, overcast and rainy weather had a greater effect on lodging resistance in indica rice compared with japonica rice. This was reflected in a greater reduction in the lignin content of the indica rice stems, which yielded a significantly lower breaking strength and bending stress.

Numerous studies have shown that the concentrations of greenhouse gases continue to rise due to human activities. The global average temperature is expected to rise by 1.8–4.0 °C by 2100^1^. This rise in temperature may lead to changes in global precipitation patterns, such as increased precipitation at high latitudes in winter, in addition to increasing the frequency of extreme weather events^2^. These climatic changes will exert a profound impact on agricultural production, including lodging resistance in rice, through changes in crop growth processes, suitable planting areas, and disaster factors.

Rice is the most important staple food crop in China and Asia. Lodging of rice stems destroys the photosynthetic capacity of the canopy, which greatly affects grain filling. This effect causes yield loss, a decline in grain quality, and increases in harvest costs, severely limiting the balanced yield increases for rice in large areas^3^. Lodging resistance in rice depends on the relationship between the weight of the upper plants and the bending load of basal internodes^4^. The lodging index (LI) is often used to evaluate lodging resistance^5^. Plant height, height of the gravitational centre (HGC), length of elongated internodes of basal stems, plumpness, culm diameter, and culm wall thickness all influence the lodging resistance capacity of rice^6^,^7^,^8^,^9^. Plant height cannot be further reduced without affecting rice yields, owing to the restriction of the photosynthetic capacity of dwarf plants^10^. Therefore, a new strategy for improving lodging resistance is to improve the stem strength of basal internodes^10^,^11^. Additionally, studies have shown that the contents of cellulose^12^, starch^13^, lignin^14^, and soluble sugars^15^ in the cell wall have a significant impact on lodging resistance in rice.

The reproductive growth stage is a critical period for the formation and plumpness of rice stems. During this period, climatic conditions have a great influence on stem strength in rice. Research has shown that low light promotes internode elongation and reduces culm wall thickness. This reduction results in fragile supporting tissue and low stem strength, thus aggravating lodging^16^. However, no study has systematically investigated how low-temperature, overcast and rainy weather affects the reproductive growth stage and rice stem strength and the associated differences between varieties.

A rare period of long low-temperature, overcast and rainy weather was encountered during the reproductive growth stage of rice in East China in 2014. In this study, we conducted experiments related to lodging on two indica hybrid rice varieties and two japonica rice varieties under the same field management conditions. The lodging resistance capacities of rice showed significant differences in 2014. Therefore, we analysed the differences in lodging resistance of rice stems from 2012 to 2014 in terms of physical properties, morphological characteristics, and chemical composition to provide a reference for research into lodging resistance in rice.

## Materials and Methods

### Comparison of climatic conditions

**Figure 1:**
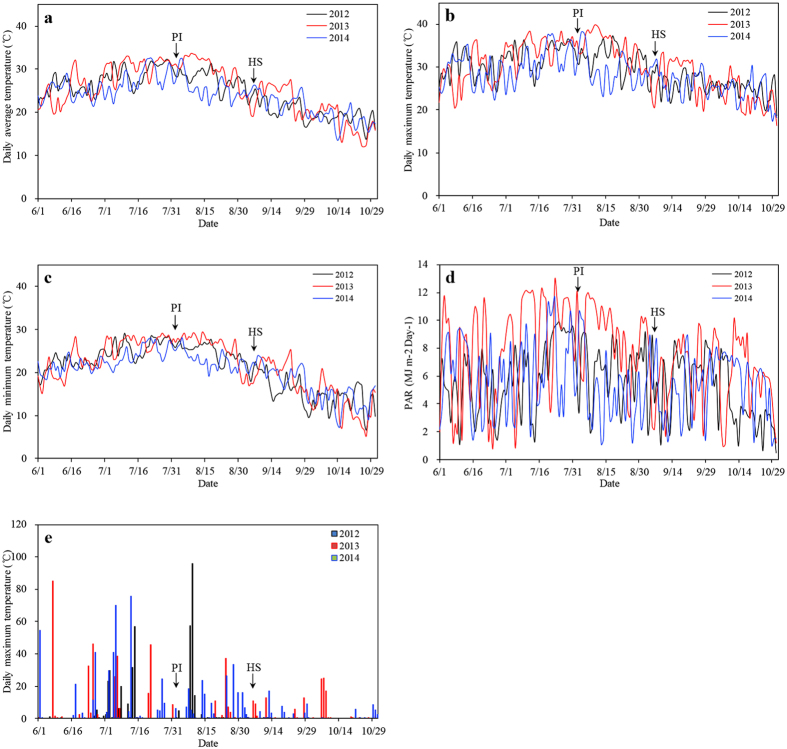
Daily average temperature (**a**), Daily maximum temperature (**b**), Daily minimum temperature (**c**), PAR (**d**) and Precipitation (**e**) during the whole rice growth stage from June to October in 2012, 2013 and 2014. Data were collected by the Danyang Meteorological station. PI and HS indicate panicle imitation stage and heading stage, respectively.

Long-term means of meteorological data from 2011 to 2015 are listed, which were analysed using growth periods of japonica rice varieties (Supplemental Table S1). Since the reproductive stage (from panicle initiation [PI] to the heading stage [HS]) is the key period for internode development, weather conditions in 2012 and 2014 were extreme, characterised by low temperature, low PAR and more rainfall, especially in 2014. As a common weather condition, 2013 was compared with 2014 to determine how extreme weather could affect rice lodging. Climatic conditions were significantly different between the two years (Fig. 1). The climatic differences are illustrated below with japonica rice as an example because the growth periods between japonica and indica rice are similar.

1. The temperature before the rice HS was lower in 2014. From the transplanting stage (TS) to panicle initiation (PI), the daily mean temperature was 3.3 °C lower in 2014 than in 2013. From the heading stage (HS) to the mature stage (MS), the daily mean temperature was approximately 1 °C difference between 2013 and 2014. The greatest difference was found from PI to HS. During this period (PI–HS), the average and maximum daily mean temperature in 2014 was 4.4 °C and 5.4 °C lower than that in 2013 respectively, but the minimum daily mean temperature was 2.0 °C higher in 2014 than that in 2013. In addition, the temperature in 2012 was almost between that in 2013 and 2014 from the TS to the MS.
2. Lower photosynthetically active radiation (PAR) was observed before the rice HS in 2014. From TS to PI, PAR was 12.0 MJ m^−2^ day^−1^ in 2012, 8.9 MJ m^−2^ day^−1^ in 2013 and 6.1 MJ m^−2^ day^−1^ in 2014. From PI to HS, the most crucial stage in internode formation, PAR, was 8.6 and 4.4 MJ m^−2^ day^−1^ in 2013 and 2014, respectively. PAR was 6.6 in 2012, which was between that of 2013 and 2014. From HS to MS, PAR was 5.3, 5.5 and 5.3 MJ m^−2^ day^−1^ in 2012, 2013 and 2014, respectively.
3. Greater rainfall was observed before the rice HS in 2014. From TS to PI in 2013, the annual rainfall was 142.8 mm, and 7 rainy days were recorded. The annual rainfall was 192.0 mm, and 12 rainy days were recorded from TS to PI in 2012. During this period in 2014, the annual rainfall was 338.6 mm, and 20 rainy days were observed. From PI to HS, the annual rainfall was 171.7 mm (with 5 rainy days recorded) in 2012 and 72.9 mm (with 6 rainy days recorded) in 2013. During this period in 2014, the annual rainfall was 185.9 mm, with 20 rainy days observed. From HS to MS, the annual rainfall and the number of rainy days in 2014 were only slightly less than their respective measurements in 2013, while the annual rainfall and the number of rainy days in 2012 was close to zero.

### Experimental materials and design

Two super hybrid indica rice varieties (lodging-resistant Yliangyou2 and lodging-susceptible IIyou084) and two japonica rice varieties (lodging-resistant Wuyunjing23 and lodging-susceptible W3668) were used as the experimental materials. Field experiments were conducted at an experimental base at Nanjing Agricultural University (Nanjing, China) on the Baolin Farm in Yanling Town, Danyang City, Jiangsu Province, China. The area is located in a subtropical monsoon climate, and the soil at the site is classified as alluvial loamy. The plough layer of the experimental soil contained the following: 21.02 g/kg organic matter, 1.12 g/kg total nitrogen, 70.60 mg/kg available nitrogen, 13.23 mg/kg available phosphorus, and 119.41 mg/kg available potassium, at a pH of 6.85. Seedlings were raised in a dry nursery using nutrition seedling trays. The seedlings were transplanted into the field, and the row spacing used for rice transplantation was 13.3 cm × 30.0 cm, with two seedlings per hole. Growth periods of japonica and indica rice from 2012 to 2014 are presented in Table 1.

Table S2 shows the nitrogen (N) application treatments used in the field experiments. The total N application was 270 kg/ha, which included a base-tiller fertilizer of 135 kg/ha (basal: tillering = 50%: 50%) and a panicle fertilizer of 135 kg/ha (PI: spikelet differentiation = 60%: 40%). Basal, tillering, PI, and spikelet differentiation fertilizers were applied 1 day before transplantation, 7 days after transplantation, at the young panicle differentiation stage, and at the second leaf (penultimate leaf) stage, respectively. The experiments were arranged in a randomized complete block design with three replications. Each plot is 24 m^2^ (4 m × 6 m) and all plots were covered with plastic films on 30 cm-wide ridges to prevent fertilizer contamination. Phosphorous was applied for basal fertilizer one day before transplantation with super-phosphate at a rate of 90 kg/ha P_2_O_5_. Potassium was applied with potassium chloride at a rate of 120 kg/ha K_2_O, 50% as basal fertilizer and 50% as panicle initiation fertilizer. Other management practices followed conventional cultivation methods.

### Field sampling and calculation methods

Thirty days after heading, 10 main stems with consistent growth were selected to measure the HGC and the length of the first and second basal internodes. The HGC was measured as follows: the fresh plant was placed on the tip of a pencil, and the fulcrum point was adjusted to achieve balance. The distance from the base to the pencil tip was defined as the HGC (cm). Next, an AIKON RX-5 digital force gauge (Company: AIKON, Japan) was used to break the midpoint of the second basal internode and measure the bending load at a distance of 8 cm between two fulcrum points. The second basal internode was then cut off from the midpoint, and a vernier calliper was used to measure the inner and outer diameters of the major and minor axes in a hollow oval stem after the removal of the leaf sheath. Physical parameters on the second basal internode were calculated using the following formulas (Ookawa, 1992)^5^: (1) Bending moment of the whole plant (WP, g.cm), WP = SL × FW, where SL and FW are the length (cm) and fresh weight (g) from the broken point to the panicle tip, respectively; (2) Breaking strength (M, g.cm), M = F × L/4, where F is the bending load of the second basal internode (kg), and L is the distance between two fulcrum points (cm); (3) Cross-section modulus (Z, mm^3^), Z = π/32 × (a_1_^3^b_1_ − a_2_^3^b_2_)/a_1_, where a_1_ and a_2_ are the outer and inner diameters along the minor axis, respectively, and b_1_ and b_2_ are the outer and inner diameters along the major axis in an oval cross-section, respectively (mm); (4) Bending stress (BS, g.mm^−2^), BS = M/Z, representing stem strength; (5) Lodging Index (LI, %), LI = WP/M, indicating susceptibility to lodging; Lodging Rate (LR, %), representing the percentage of lodging plants in all the plants. The second basal internodes were separated into culms and leaf sheaths, deactivated at 105 °C for 30 min and dried at 80 °C to measure dry weight. The dried samples were ground into a fine powder and passed through a 100-mesh sieve before chemical composition analysis.

### Chemical composition analysis of rice stems

Non-structural carbohydrate (NSC) content was determined according to the method of Yoshida *et al*.^17^. For structural carbohydrate (SC) content analysis, cellulose content was determined using the method of Updegraff *et al*.^18^ and Zhang *et al*.^15^. Lignin content was determined in accordance with Ishimaru *et al*.^13^.

### Statistical analysis

Data were processed using Microsoft Excel 2003. Analysis of variance was performed using SPSS 17.0 for windows based on the significant difference test (Duncan) at 0.05 or 0.01 probability level. Plots were generated using OriginPro 8.0.

## Results

### Rice yield

Table 2 summarises the yield and yield components of different varieties. However, the interaction between years and varieties was only observed in panicles and 1000-grain weight. The lodging-resistant varieties Yliangyou2 and Wuyunjing23 had 12% and 31% higher yields, respectively, than the lodging-susceptible varieties IIyou084 and W3668. Although no significant difference was discovered among the years, effects still existed by comparing the two most extreme weather conditions in 2013 and 2014. For indica rice varieties, interannual comparisons showed that the grain yields of Yliangyou2 and IIyou084 were reduced by 10.9% and 10.1%, respectively, in 2014 compared with 2013. The lower yields in 2014 were due to reductions in the numbers of productive panicles and spikelets per panicle, which resulted in significantly fewer spikelets (15.0% and 18.5% fewer spikelets were observed in Yliangyou2 and IIyou084, respectively). For japonica rice varieties, Wuyunjing23 and W3668 yields increased by 3.4% and 5.4%, respectively, in 2014 compared with 2013. Although a reduction in productive panicles resulted in fewer spikelets, the seed-setting rate and the 1000-grain weight both increased in the japonica rice varieties (the seed setting rate of Wuyunjing23 and W3668increased by 5.4% and 3.4%, and the 1000-grain weight increased by 3.1% and 5.8%, respectively), which were likely the main reasons for the observed yield increases. Moreover, due to the higher seed-setting rate and 1000-grain weight in 2012 than in 2013, the yields were even higher in IIyou084 and Wuyunjing23, which might have been the result of good weather conditions during the HS to the MS in 2012.

### Physical properties

Significant differences existed in nearly all parameters except LR regarding the effect of years, indicating that low-temperature, overcast and rainy weather does have an apparent influence on rice lodging (Table 3). Although higher lodging Rate (LR) was observed in 2014 especially in japonica varieties, it is still not rigorous to measure lodging resistance by using LR since LR is largely affected by weather conditions in late period instead of revealing lodging resistance of plants themselves like LI. LI significantly increased in 2012 and 2014 compared with 2013. However, an analysis of the effects of WP and M (WP and M are calculation factors of LI) revealed that different varieties during different years had different factors that caused LI to increase. Compared with 2013, the LI values of Yliangyou2, IIyou084, Wuyunjing23 and W3668 increased by 14.4%, 13.7%, 32.7%, and 23.3% in 2012 and by 19.4%, 33.8%, 56.3%, and 26.2% in 2014, respectively (Table 3). With regard to the source of the LI increase, WP and M were significantly reduced for both of the indica varieties, while the reduction in M was greater. For the two japonica varieties, the increase in LI was mainly due to a reduction in M, as WP even increased in 2012 and 2014. The indica varieties exhibited no obvious differences in SL among the years, whereas FW was significantly reduced, resulting in a lower WP. However, due to a significant increase in SL, the japonica varieties had no reduction in WP.

Compared with 2013, the M values of Yliangyou2, IIyou084, Wuyunjing23 and W3668 were reduced by 21.0%, 13.6%, 3.1%, and 6.6% in 2012 and by 33.1%, 34.8%, 19.4%, and 22.2% in 2014, respectively. Among the different varieties, indica rice exhibited a more obvious reduction in M. This was mainly due to a significant reduction in BS. In 2014, BS was reduced by 20.4% and 39.8% in Yliangyou2 and IIyou084, but by only 4.3% and 13.7% in Wuyunjing23 and W3668, respectively. However, in 2012, BS was reduced by 12.9%, 29.0% for indica varieties and by 17.1%, 31.3% for japonica varieties, which did not show much difference. The Z values showed inconsistent changes between varieties. A significant difference among years was observed in the japonica varieties, but not in the indica varieties. The results showed that low-temperature, overcast and rainy weather could increase the risks of rice lodging and had a greater effect on indica varieties than on japonica varieties in terms of two stem strength parameters (BS and M).

### Configuration of the internodes and HGC

**Figure 2:**
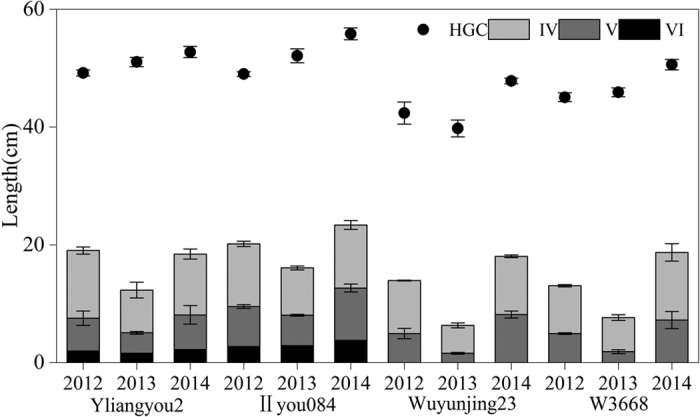
Configuration of internodes of different varieties from 2012 to 2014. The uppermost internode was named I; others were followed by II–VI.

Figure 2 presents the configuration of the internodes and HGC in different varieties. On average, indica rice was taller than japonica rice. The HGC and the lengths of internodes VI, V and IV significantly differed among the years (Table 4). Compared with 2013, the lengths of the first, second and third basal internodes in Yliangyou2 increased by 38.5%, 70.8% and 42.3 in 2014, respectively. While in IIyou084, these increases were 31.5%, 71.5% and 33.4%. The HGC values for Yliangyou2 and IIyou084 were 3.3% and 7.1% greater in 2014 compared with 2013, respectively. For japonica rice, the lengths of the first and second basal internodes increased significantly. Compared to 2013, the first and second basal internodes increased by 413.0% and 108.8%, respectively, in Wuyunjing23 in 2014. The first and second basal internodes of W3668 increased by 288.2% and 98.7%, respectively, over the same period. These differences were extreme and significant. The HGC values for Wuyunjing23 and W3668 were 20.3% and 10.3% greater in 2014 compared to 2013. However, the increase in internode lengths of all varieties in 2012 was less than in 2014. Moreover, the HGC in 2012 was even lower than that in 2014, which was also reflected by an interaction effect between years and varieties on HGC (Table 4).

### Morphological characteristics of basal internodes

As seen in Table 5, there was no interaction between varieties and years. It also shows that compared with 2013, the dry weight of basal culms of Yliangyou2, IIyou084, Wuyunjing23 and W3668 was significantly reduced by 21.0%, 26.7%, 15.5% and 36.0% in 2014, respectively. The culm diameter in Yliangyou2, IIyou084, Wuyunjing23 and W3668 decreased by 9.1%, 4.9%, 3.4% and 5.2% in 2014 compared with 2013. However, we did not observe such a large reduction in 2012. Although no significant difference was observed, indica rice varieties exhibited downward trend in culm wall thickness from 2013 to 2014 while no reduction in culm wall thickness were presented in japonica rice varieties between the two years. In contrast, dry weight of the basal leaf sheath exhibited an opposite trend, reaching the minimum in 2013, except in japonica W3668.

### Carbohydrates in basal culms and leaf sheaths

In addition, to analyse whether the carbohydrates in the basal culms and leaf sheaths were altered under extreme weather conditions, NSC, cellulose, and lignin contents were measured (Table 6). Significant effects existed in the NSC and lignin contents in the basal stems among the years. However, the NSC contents did not show a consistent trend in all varieties. Compared with 2013, the lignin content was lower in 2012 and 2014, especially affected by the extreme weather in 2014. The lignin content of Yliangyou2 and IIyou084 was reduced significantly by 45.0% and 41.0% and the total content of SC (lignin and cellulose) was reduced by 21.6% and 9.8% in 2014, respectively, compared with 2013. For the japonica rice varieties Wuyunjing23 and W3668, the cellulose content decreased significantly by 17.9% and 25.4%, the lignin content decreased by 2.3% and 19.1%, and the total content of SC (lignin and cellulose) decreased by 12.8% and 23.4 %in Wuyunjing23 and W3668, respectively. Moreover, the interaction between years and varieties was observed in the effect of lignin contents in the basal culms. The lignin content in the basal stems showed greater interannual differences in indica rice varieties compared with japonica rice varieties. Interestingly, leaf sheaths had relatively high contents of NSC, cellulose and lignin, especially in the indica varieties.

## Discussion

### Low-temperature, overcast and rainy weather decreased the rice lodging resistance

In this study, the climate condition in 2013 was normal when compared with the average value of multi-year. However, the daily mean temperature and PAR were lower before the rice HS in 2012 and 2014 compared with 2013, especially for 2014. Moreover, a longer period of low-temperature, overcast and rainy weather was encountered in 2012 and 2014. There was no significant difference in PAR, temperature and rainfall after the HS between 2013 and 2014, but there was nearly no precipitation in 2012 (Fig. 1 and Supplemental Table S1). The results showed that low-temperature, overcast and rainy weather exerted a profound impact on lodging resistance in rice stems, with a significant increase in the LI (Table 3). TS to PI occurs during the vegetative growth period of rice plants, while PI to HS is critical for elongation and the plumpness of stem internodes. We inferred that the LI increase in 2012 and 2014 was mainly due to prolonged low-temperature, overcast and rainy weather from PI to HS, the reproductive growth period of rice.

### Indica and japonica rice responded differently to extreme weather

Genotype is a key factor in determining rice lodging, and variations are often observed between rice varieties^6^,^19^. The reason for the LI increase varied in the indica and japonica varieties. The results showed that FW was markedly reduced in indica rice, but not in japonica rice. The number of spikelets per panicle also showed the same trend, decreased significantly in indica rice, but a little in japonica rice (Table 2). For japonica rice, FW did not decrease and SL increased, which resulted in a significant increase in WP. This phenomenon explained the remarkable increase in the LI of the two japonica varieties. Although the WP of the indica rice decreased while that of japonica rice increased, the M values of both types of rice were reduced. In addition, this reduction in M as well as in BS was more obvious in indica rice than in japonica rice (Table 3). These results lead us to infer that the yield and lodging resistance of indica rice are more significantly affected by low-temperature, overcast and rainy weather. In addition, elongated basal internodes and increased plant height were much more obviously observed in japonica rice, while the lignin content in the basal internodes showed a greater interannual difference in the indica rice varieties compared with japonica rice. This phenomenon might explain why greater reductions in M and BS were observed in the two indica rice varieties but not in the two japonica rice varieties.

### Light may be the main cause of rice lodging under low-temperature, overcast and rainy weather

Low light and shading can cause excessive growth in plants, with elongated basal internodes, higher HGC, and lower lodging resistance^20^,^21^,^22^. Such reduction of lodging resistance has also been observed in maize^23^ and soybean^24^. A similar phenomenon was observed in this study. Weather changes had an obvious impact on many morphological characteristics, such as HGC and length of the elongated internodes of basal stems, which may be induced by an increased gibberellin content according to previous studies^25^,^26^. In addition, the stem strength of rice stems is also closely related to their plumpness. The plump components of rice stems mainly include NSC (e.g., soluble sugar and starch) and SC (e.g., cellulose and lignin). Scholars have suggested that stems contain a high content of starch. During the grain filling stage after heading, the starch retained in stems is used to improve lodging resistance in plants^27^. However, Zhang *et al*.^28^ reported that starch and soluble sugar contents in stems have little relationship with bending load. Instead, bending load is mainly associated with cellulose and lignin contents in basal stems, and greater cellulose and lignin contents result in greater stem strength^15^. Former research has indicated that light intensity can promote the expression of genes related to Lignin biosynthesis, thus increasing the lignin content^29^,^30^. Here, an obvious reduction in lignin content was also observed, and such a decrease was possibly due to relatively low PAR in 2012 or 2014. More in-depth, light quantity (PAR) was associated with stem traits associated with lodging, while light quality (F:FR) played a role in determining rooting traits that affect the anchorage capacity of wheat plants^31^,^32^. However, in our present study, the comprehensive effect of temperature, light and rainfall was emphasised. Further investigation is necessary to determine the separated effect of single factors on rice lodging and to compare whether it is the same.

### Potential value of understanding the effect of low-temperature, overcast and rainy weather on rice lodging resistance

Frequent extreme weather will bring great risks to rice production, so it is meaningful to determine the potential effect of such weather on rice growth and development. Recently, multiple studies have focused on whether cultivation measures have an impact on rice lodging, such as planting density^33^ or nitrogen application rates^34^,^35^,^36^. In addition, plant growth regulators can be involved in the formation of stem morphology and substance accumulation, thus affecting the mechanical strength and lodging resistance of stems. Gibberellins and paclobutrazol or uniconazole have shown significant and rapid effects on rice lodging^37^,^38^. Our results clarified that low-temperature, overcast and rainy weather reduced the lodging resistance of rice and have a negative effect on yields, and the response of rice to this extreme weather was different between japonica and indica varieties. This is helpful for us to choose the appropriate measures to avoid the rice lodging and ensure the rice high yield when the extreme weather is encountered.

## Additional Information

**How to cite this article:** Weng, F. *et al*. Impact of low-temperature, overcast and rainy weather during the reproductive growth stage on lodging resistance of rice. *Sci. Rep.* **7**, 46596; doi: 10.1038/srep46596 (2017).

**Publisher's note:** Springer Nature remains neutral with regard to jurisdictional claims in published maps and institutional affiliations.

## Supplementary Material

Supplementary Tables

## Figures and Tables

**Table 1:** Specific dates of the growth stages for japonica and indica rice from 2012 to 2014.

Varieties	Growth stage	2012	2013	2014
Japonica rice (Wuyunjing23 and W3668)	SS	05/26	05/27	05/27
	TS	06/25	06/27	06/21
	PI	08/06	08/07	08/06
	HS	09/05	09/05	09/10
	MS	10/31	10/31	11/04
Indica rice (Yliangyou2 and IIyou084)	SS	05/26	05/27	05/27
	TS	06/25	06/27	06/21
	PI	08/02	08/05	08/10
	HS	09/02	09/01	09/04
	MS	10/30	10/31	11/04

The values in the table represent ‘month/day’.

SS: Seeding stage; TS: Transplanting stage; PI: Panicle initiation; HS: Heading stage; MS: Mature stage.

**Table 2:** Yield and its components of different rice varieties in 2012, 2013 and 2014.

Varieties		Panicles (×10^4^ hm^−2^)	Spikelets per panicle	Spikelets (×10^5^ hm^−2^)	Seed-setting rate (%)	1000-grain weight (g)	Yield (t hm^−2^)
Yliangyou2	2012	300.1a	164.7b	4935.5b	91.2a	26.5a	12.0a
	2013	297.8a	203.9a	6060.7a	88.0a	24.1b	12.9a
	2014	280.7a	183.5ab	5152.3b	85.7a	26.1a	11.5a
IIyou084	2012	281.2a	152.7a	4297.0a	94.9a	28.7ab	11.7a
	2013	311.2a	151.7a	4648.2a	83.8b	28.1b	10.9a
	2014	281.4a	135.0a	3787.8a	87.8b	29.5a	9.8a
Wuyunjing23	2012	333.7a	120.4a	4005.6b	94.9a	32.3a	12.3a
	2013	371.7a	121.6a	4517.8a	87.7b	29.2b	11.6a
	2014	343.2a	126.4a	4324.6ab	92.4ab	30.1b	12.0a
W3668	2012	382.6b	99.1a	3792.4b	92.7a	24.5a	8.6a
	2013	447.6a	105.7a	4747.9a	86.0a	22.6b	9.2a
	2014	428.1a	106.6a	4561.3ab	88.9a	23.9ab	9.7a
**Significance**							
Y		11.7**	1.6 ns	9.3**	15.0**	42.0**	0.7 ns
V		112.6**	41.8**	14.3**	1.9 ns	286.5**	17.2**
Y*V		2.6*	1.4 ns	1.5 ns	1.2 ns	4.6**	1.7 ns

Y, year; V, varieties. The same holds for the tables below.

Difference was compared for each variety within 3 years. Values labelled with different letters are significantly different at the 0.05 level. The same holds for the tables below.

**F-values significant at the 0.01 probability levels. *F-values significant at the 0.05 probability levels. ns, F-values not significant at the 0.05 probability levels. The same holds for the tables below.

**Table 3:** The characteristics related to lodging among different varieties from 2012 to 2014.

Varieties		WP (g.cm)	FW (g)	SL (cm)	M (g.cm)	BS (g.mm^−2^)	Z (mm^3^)	LI (%)	LR (%)
Yliangyou2	2012	2890.2a	25.4b	113.7a	1752.6b	997.3b	18.0a	164.9a	29.8a
	2013	3193.2a	28.6a	111.8a	2219.5a	1144.8a	19.5a	144.1b	1.37b
	2014	2551.7b	23.0b	110.9a	1485.4c	911.8b	16.4a	172.0a	0.31b
IIyou084	2012	2992.2a	26.6a	112.4a	1490.7a	644.8ab	23.1a	201.0b	75.2a
	2013	3032.5a	27.5a	110.4ab	1726.0a	908.2a	19.8a	176.8b	64.6a
	2014	2663.1a	24.7a	107.8b	1125.6b	546.3b	20.7a	236.6a	61.0a
Wuyunjing23	2012	1937.6a	20.5a	94.4b	1896.6a	1922.7b	9.9a	102.6a	0.9b
	2013	1508.8b	18.2a	82.7c	1956.9a	2320.9a	8.4b	77.3b	0b
	2014	1891.7a	18.9a	99.8a	1576.3b	2220.8ab	7.1c	120.8a	10.6a
W3668	2012	1536.9a	15.1a	101.6b	772.9a	1149.4c	6.7a	200.4a	61.4a
	2013	1343.0a	14.3ab	94.0c	827.5a	1672.4a	5.0b	162.5b	76.8a
	2014	1320.9a	12.3b	107.1a	643.5b	1443.8b	4.5c	205.0a	91.2a
**Significance**									
Y		7.8**	12.0**	48.7*	44.0**	21.3**	5.5*	36.7**	0.5 ns
V		241.4**	175.5**	230.4**	147.1**	211.6**	178.2**	121.8**	45.5**
Y*V		7.2**	3.5*	22.9**	3.5*	2.5 ns	1.3 ns	1.3 ns	2.0 ns

WP: Bending moment of the whole plant (g.cm); FW: The fresh weight (g) from the broken point to the panicle tip; SL: The length (cm) from the broken point to the panicle tip; M: Breaking strength (g.cm); BS: Bending stress (g. mm^−2^); Z: Cross-section modulus (mm^3^); LI: lodging Index (%); LR: lodging Rate (%).

**Table 4:** Analysis of variance of HGC (height of the gravitational centre) and the internode lengths.

Significance	HGC	the VI internode lengths	the V internode lengths	the IV internode lengths
Y	99.1**	107.0**	92.1**	4.7*
V	146.6**	14.6**	15.1**	29.8**
Y*V	7.4**	6.4**	4.1**	1.1 ns

The values for the IV internode lengths were only calculated in Yliangyou2 and IIyou084.

**Table 5:** Morphological characteristics of the basal internodes among the different varieties.

Varieties		Dry weight of the basal culm (mg/cm)	Dry weight of the basal leaf sheath (mg/cm)	Culm diameter (mm)	Culm thickness (mm)
Yliangyou2	2012	29.8a	17.8ab	6.5ab	1.03a
	2013	30.6a	13.9b	6.7a	1.01a
	2014	24.2b	18.8a	6.1b	0.97a
IIyou084	2012	27.0a	8.4a	7.3a	0.98a
	2013	28.4a	6.9a	7.2ab	0.94ab
	2014	20.8b	10.6a	6.8b	0.91b
Wuyunjing23	2012	18.3a	23.8a	4.8b	0.80a
	2013	17.8a	20.9c	5.0a	0.82a
	2014	15.0b	21.8b	4.8b	0.82a
W3668	2012	15.0b	14.8a	4.1a	0.65a
	2013	17.7a	13.2a	4.3a	0.64a
	2014	11.3c	12.1a	4.0a	0.65a
**Significance**					
Y		30.6**	4.2*	9.5**	1.0 ns
V		102.0**	58.5**	467.6**	80.0**
Y*V		1.1 ns	1.4 ns	1.9 ns	0.6 ns

**Table 6:** NSC, cellulose, lignin content and total content of SC (cellulose and lignin) in the basal internode culms and leaf sheaths among the different varieties.

		Culm (mg/cm)				Leaf sheath (mg/cm)			
Varieties		NSC	Cellulose	Lignin	SC	NSC	Cellulose	Lignin	SC
Yliangyou2	2012	2.59b	7.24a	3.20b	10.44a	1.67ab	3.90b	2.01a	5.44ab
	2013	3.50b	7.62a	4.53a	12.15a	1.02b	3.59b	1.84a	5.43b
	2014	5.09a	7.04a	2.49b	9.54a	2.24a	5.49a	2.01a	7.50a
IIyou084	2012	1.41b	5.75a	3.87a	9.62a	0.51b	1.66a	1.28a	2.94a
	2013	7.13a	5.23a	3.71a	8.94a	0.47b	1.93a	1.06a	2.99a
	2014	4.00ab	5.87a	2.19b	8.07a	1.17a	2.55a	1.42a	3.97a
Wuyunjing23	2012	1.74a	4.46b	2.78a	7.24b	2.22a	5.94a	3.91b	9.85b
	2013	0.75b	5.48a	2.64a	8.12a	0.77b	6.64a	3.91b	10.55ab
	2014	0.75b	4.50b	2.58a	7.08b	0.90b	6.62a	4.89a	11.50a
W3668	2012	1.86b	3.89ab	1.75a	5.64ab	0.83a	3.10a	2.03a	5.12a
	2013	3.77a	4.49a	2.04a	6.54a	0.66a	4.37a	2.43a	6.81a
	2014	1.11b	3.35b	1.65a	5.00b	0.63a	3.74a	2.69a	6.43a
**Significance**									
Y		5.3*	1.1 ns	47.9**	6.6**	11.0**	4.3*	6.8**	5.2*
V		8.9**	24.8**	72.3**	38.2**	17.6**	46.3**	128.5**	71.6**
Y*V		4.1**	0.7 ns	14.4**	0.9 ns	7.3**	1.5 ns	1.9 ns	0.8 ns
