# Supplementary material for: Impact of low-temperature, overcast and rainy weather during the reproductive growth stage on lodging resistance of rice
Source: Sci Rep. 2017 Apr 19;7:46596. doi: 10.1038/srep46596 (PMC5395946; doi:10.1038/srep46596)
Supplement: Supplementary Tables [file srep46596-s1.doc]

**Impact of low-temperature, overcast and rainy weather during the reproductive growth stage on lodging resistance of rice**

**Fei Weng^1^, Wujun Zhang^1, 2^, Xiaoran Wu^1^, Xia Xu^1^, Yanfeng Ding^1^, Ganghua Li^1*^, Zhenghui Liu^1^, Shaohua Wang^1^**

^1^National Engineering and Technology Center for Information Agriculture/Key Laboratory of Crop Physiology and Ecology in Southern China/Jiangsu Collaborative Innovation Center for Modern Crop Production, Nanjing Agricultural University, Nanjing 210095, China

^2^ Chongqing Academy of Agricultural Sciences/Chongqing Ratooning Rice Research Center, Chongqing402160, China

**^*^Corresponding author:**

Mailing address: Agronomy College, NAU; 1^#^Weigang Street, Nanjing, China, 210095

Tel.: 086-25-84396475; Fax No.: 086-25-84396302

Gang-Hua LI, E-mail: lgh@njau.edu.cn

Table S1

Average temperature, maximum and minimum temperatures, photosynthetic active radiation (PAR), precipitation and days of rainfall at Danyang from 2011 and 2015.

| Growth stages | *TS –PI* | | | | |  | *PI-HS* | | | | |  | *HS-MS* | | | | |
| --- | --- | --- | --- | --- | --- | --- | --- | --- | --- | --- | --- | --- | --- | --- | --- | --- | --- |
|  | 2011 | 2012 | 2013 | 2014 | 2015 |  | 2011 | 2012 | 2013 | 2014 | 2015 |  | 2011 | 2012 | 2013 | 2014 | 2015 |
| Average temperature (°C) | 27.8 | 29.0 | 30.1 | 26.8 | 26.9 |  | 26.7 | 27.8 | 29 | 24.6 | 26.1 |  | 19.4 | 19.6 | 20.4 | 19.4 | 20.7 |
| Maximum temperature (°C) | 32.2 | 32.4 | 33.1 | 32.6 | 32.1 |  | 30.8 | 31.4 | 33.7 | 28.3 | 27.7 |  | 28.7 | 27.5 | 27.5 | 25.7 | 26.4 |
| Minimum temperature (°C) | 21.8 | 23.0 | 23.7 | 21.4 | 20.2 |  | 20.5 | 22.8 | 19.4 | 21.4 | 24.1 |  | 12.3 | 11.7 | 12.0 | 9.9 | 13.8 |
| PAR (MJ m^-2^ day^-1^) | 5.3 | 12.0 | 8.9 | 6.1 | 7.1 |  | 5.2 | 6.6 | 8.6 | 4.4 | 8.5 |  | 3.6 | 5.3 | 5.5 | 5.3 | 7.5 |
| Precipitation (mm) | 260.2 | 192.0 | 142.8 | 338.6 | 287.5 |  | 249.6 | 171.7 | 72.9 | 185.9 | 0 |  | 41.8 | 1.0 | 113.3 | 72.1 | 38.4 |
| Days of rainfall | 21 | 12 | 7 | 20 | 20 |  | 21 | 5 | 6 | 20 | 0 |  | 11 | 1 | 12 | 19 | 12 |

The data in the table were calculated as the daily average. PAR: Photosynthetically active radiation; TS: Transplanting stage; PI: Panicle initiation; HS: Heading stage; MS: Mature stage.

Table S2

Nitrogen (N) application treatments during the field experiments (kg N ha^-1^).

| Nitrogen application  (kg N ha^-1^) | Base-tiller fertilizer | |  | Panicle fertilizer | |
| --- | --- | --- | --- | --- | --- |
|  | Basal | Tillering |  | Panicle initiation | Spikelet differentiation |
| 270 | 67.5 | 67.5 |  | 81 | 54 |
